# Effects of Tick Infestation on Milk Yield, Blood Biochemistry, Hematology, and the Overall Health of Dairy Cows

**DOI:** 10.3390/pathogens14090883

**Published:** 2025-09-03

**Authors:** Mona Al-Shammari, Ibrahim O. Alanazi, Mohammad Alzahrani, Samiah Alotaibi, Nora Alkahtani, Almaha Alaqil, Ebtesam Al-Olayan

**Affiliations:** 1 Zoology Department, College of Science, King Saud University, Riyadh 11451, Saudi Arabia; 443204468@student.ksu.edu.sa (M.A.-S.); samiahalotaibi96@gmail.com (S.A.); alkahtani.ns@gmail.com (N.A.);; 2 Healthy Aging Research Institute, Health Sector, King Abdulaziz City for Science and Technology, Riyadh 11451, Saudi Arabia; ialenazi@kacst.gov.sa; 3 Institute of Advanced Agricultural and Food Technologies, King Abdulaziz City for Science and Technology, Riyadh 11442, Saudi Arabia; mzahrani@kacst.edu.sa

**Keywords:** *Hyalomma anatolicum*, ticks, cytochrome c oxidase subunit I, molecular and morphological identification, milk production, Saudi Arabia

## Abstract

Tick infestation represents a significant constraint on livestock productivity in Saudi Arabia; however, there remains a substantial gap in research addressing tick species diversity, distribution, and their direct effects on milk production. This study aimed to morphologically and molecularly identify tick species infesting dairy cattle, quantify the impact of tick infestation on milk yield and composition, and contribute to the limited understanding of tick ecology and its economic implications in the region. Ticks were collected from infested cows and identified morphologically using taxonomic keys. Molecular identification was performed via PCR amplification of *the mitochondrial cytochrome c oxidase subunit I (COI)* gene. Milk production and quality parameters were assessed in tick-infested and healthy cows in Hafar Al-Batin, Eastern Saudi Arabia. Morphological and genetic analyses confirmed *Hyalomma anatolicum* as the predominant tick species in the study area, with COI sequences showing high similarity to regional isolates. Tick-infested cows exhibited substantial reductions in milk yield, fat, calcium, and potassium levels, indicating significant metabolic disruptions. Blood biochemical analysis revealed elevated levels of liver enzymes [aspartate aminotransferase (AST) increased by 238.6%, gamma-glutamyl transferase (GGT) by 155.7%], renal markers [creatinine increased by 788.9%, urea by 130.0%], and electrolyte imbalances [serum calcium decreased by 39.5%, potassium by 45.2%]. Hematological findings included increased white blood cell (WBC) and red blood cell (RBC) counts by 44.9% and 124.7%, respectively, along with a 53.1% decrease in hemoglobin (HGB), suggesting a systemic inflammatory response and possible anemia. This study is among the first to genetically confirm the presence of *H. anatolicum* in Hafar Al-Batin using molecular tools, thereby enhancing the accuracy of species-level identification and highlighting the physiological impact of tick burden on dairy productivity.

## 1. Introduction

Ticks are the most economically important ectoparasites affecting livestock health and productivity, especially in tropical and subtropical regions [1]. The most common genus in Saudi Arabia is *Hyalomma* spp., which is widely distributed in arid areas where environmental conditions favor tick proliferation. Dairy cows in such regions are at high risk of tick infestations, which not only cause injury due to blood feeding but also exert indirect impacts by transmitting tick-borne diseases such as those caused by *Theileria*, *Babesia*, and *Anaplasma* species [2]. These infections result in systemic illness, reduced weight gain, and significant losses in milk yield and quality [3].

Ticks belong to the subclass Acari, class Arachnida, and are grouped into two families of veterinary importance: *Ixodidae* (hard ticks) and *Argasidae* (soft ticks). Among these, *Ixodidae* is considered more medically and economically significant due to its widespread distribution and critical role in disease transmission [4]. The dominant tick genera infesting livestock in Saudi Arabia include *Hyalomma, Rhipicephalus, Amblyomma*, and *Boophilus* [5]. *Hyalomma anatolicum* and *H. dromedarii* are particularly common in cattle and camels [6]. *H. anatolicum* is a known vector for *Theileria annulata*, the causative agent of tropical theileriosis in cattle, while *H. dromedarii* is generally associated with camels and is capable of transmitting *Coxiella burnetii* and *Anaplasma* spp. [7].

Accurate tick species identification is essential for effective disease surveillance and control programs. Classical morphological tools, based on external characteristics such as scutal patterns, genital aperture, and coxal structures, are commonly employed. However, significant intraspecific variation and morphological similarities between closely related species make these methods challenging and sometimes unreliable [8]. Molecular tools, including polymerase chain reaction (PCR) and DNA sequencing—particularly targeting the mitochondrial *cytochrome c oxidase subunit I* (*COI*) gene—have improved species-level accuracy and have been successfully applied in Saudi Arabia to identify *Hyalomma* spp. infesting camels and cattle [4,9].

In dairy cattle, tick infestations not only cause visible skin damage and discomfort but also result in substantial economic losses. Blood-sucking ticks contribute to anemia, stress, and immunosuppression, leading to reduced milk yield, lower milk quality, increased treatment costs, and potential culling of chronically infected animals [10,11]. Milk quality is a critical indicator of animal welfare and farm profitability [12].

Furthermore, minerals such as calcium and phosphorus (Ca and P) are essential for milk secretion and skeletal integrity, while magnesium and potassium (Mg and K) are crucial for nerve transmission and enzyme activation. Several studies have reported that parasitic infestations may disrupt the metabolic functions required for normal milk production [13,14]. Notably, biochemical disturbances such as hypocalcemia (low calcium), hypokalemia (low potassium), and elevated liver enzymes—including aspartate aminotransferase (AST) and alanine transaminase (ALT)—have been documented in infested animals [15]. Additionally, an increased somatic cell count (SCC), a key indicator of udder health, is frequently observed [16], signifying inflammatory or infectious processes [17,18]. These physiological disruptions can negatively impact mammary gland function and alter milk composition, including fat and protein content [19,20].

Despite the evident impact of tick infestations on dairy production, comprehensive studies correlating blood and milk parameters with tick burden remain scarce in Saudi Arabia. Current literature tends to emphasize regional tick distribution and pathogen detection without integrating animal productivity data. Addressing this research gap is essential for designing targeted control strategies and minimizing financial losses in the dairy sector.

The influence of tick infestation on milk yield, milk composition (fat, protein, calcium, and potassium), SCC, as well as blood indices (selected hematological and biochemical parameters) is studied in this paper. The study will be useful to provide scientific evidence on the cross-effects of ectoparasite infestation during dairying under Saudi Arabian conditions by studying the physiological as well as productive responses in dairy cows over 8 weeks.

## 2. Materials and Methods

### 2.1. Study Area

The study was conducted at Haleeba Dairy Farm, located in Hafar Al-Batin, Eastern Saudi Arabia (Latitude 28.3468° N, Longitude 45.9235° E), during March and April 2024. The region is characterized by an arid desert climate, with average daily temperatures ranging between 35 °C and 40 °C, and relative humidity levels between 8% and 12%, according to the Saudi National Center for Meteorology (https://ncm.gov.sa, accessed on 1 May 2025). To contextualize regional occurrence, we compiled published records of Hyalomma distribution in Saudi Arabia and the UAE (Supplementary Table S1). 

### 2.2. Animal Selection, Housing, and Study Design

Thirty Jersey lactating adult dairy cows (4–6 years of age and 550–570 kg body weight) were randomly selected from a free-grazing herd that had routine exposure to ticks under natural field conditions. Prior to the start of the experiment, all animals underwent clinical examination by a licensed veterinarian to assess their tick infestation status. The cows in this study did not exhibit any clinical manifestation of bacterial, viral, helminthic, or protozoal infections. Based on this assessment, cows were divided into two groups:

Group I (Tick Infested Group): 15 cows presented ticks on the body, especially at the ears, udder, axilla, and tail. Ticks were recognized by direct visual assay by a veterinarian. Group II (Negative control): 15 healthy cows with no evidence of ticks or infestation.

To ensure standardized conditions and prevent further tick exposure during the experimental period, all selected cows were transferred to individual pens and maintained under controlled conditions. Each pen was physically isolated, and strict hygiene and biosecurity protocols were implemented to minimize the risk of cross-contamination.

Tick control measures, including manual removal, acaricide application, and regular inspection, were applied exclusively to the healthy control group to preserve their tick-free status. In contrast, the tick-infested group received no treatment during the study in order to monitor the natural progression of tick burden and its physiological impact over time.

All cows were fed a standard diet of hay and silage with ad libitum access to water. Feeding, housing, and milking practices were identical across groups to minimize confounding variables.

### 2.3. Tick Collection and Species Identification

Ticks were collected daily over an eight-week period (Supplementary Table S2, per-cow daily counts) by examining the entire body surface of each cow in the tick-infested group (Group I), with particular attention to predilection sites such as the ears, neck, dewlap, axillae, udder, perineum, and tail. Adult ticks were carefully removed using blunt forceps and placed into sterile containers filled with 70% ethanol. Each container was clearly labeled with the date and time of collection. The collected ticks were transported daily to the laboratory for morphological species identification (Daily tick counts per cow). In total, 250 adult were recovered across the study (Supplementary Table S3) were collected from Group I and examined under a stereomicroscope using standard taxonomic keys and morphological descriptions [21,22,23,24]. No ticks were detected or removed from the control group (Group II) during the study period.

### 2.4. Molecular Identification

#### 2.4.1. DNA Extraction from Ticks

Six adult ticks were randomly selected from six different cows (out of the fifteen tick-infested animals) for molecular identification. Before DNA extraction, ticks were thoroughly washed to eliminate surface contaminants and microorganisms. Each tick was immersed in a 5% sodium hypochlorite solution for 5 min, followed by five consecutive washes in deionized distilled water, each lasting 5 min. After the final rinse, the ticks were preserved in 70% ethanol according to Binetruy et al. (2019) and Hoffman et al. (2020) [25,26], and the collection tubes were labeled with the corresponding date and cow number.

In the laboratory, each tick was manually crushed, and genomic DNA was extracted using the DNeasy Blood & Tissue Kit (Qiagen, Hilden, Germany), following the manufacturer’s protocol. The extracted DNA was stored at −20 °C until further analysis.

#### 2.4.2. PCR Amplification of the DNA COI Gene

For tick identification, primers targeting the *COI* region of mitochondrial DNA were used as described by [27]. The primer sequences are as follows. LCO1490 (Forward): 5′-GGTCAACAAATCATAAAGATATTGG-3′; HCO2198 (Reverse): 5′-TAAACTTCAGGGTGACCAAAAAATCA-3′.

PCR amplification was performed in a 25 μL reaction mixture containing approximately 80 ng of extracted tick DNA, 10 pmol of each primer, and 12.5 μL of 2× PCR Master Mix (Promega, Madison, WI, USA). Reactions were carried out in a Multigene™ thermocycler (Labnet International, Inc., Edison, NJ, USA) under the following cycling conditions: an initial denaturation at 98 °C for 3 min; followed by 35 cycles of denaturation at 95 °C for 30 s, annealing at 58 °C for 30 s, and elongation at 72 °C for 1 min; and a final extension at 72 °C for 5 min.

PCR products were analyzed by electrophoresis using 2% agarose gel stained with ethidium bromide and visualized under a UV transilluminator.

#### 2.4.3. DNA Sequencing and GenBank Accession Numbers and Phylogenetic Analysis

To identify the species of the collected ticks, six of the amplified PCR products were sent for sequencing at Life Technologies (USA). Sequence alignment and similarity analysis were performed using the NCBI BLAST tool (2.16.0). Phylogenetic trees were constructed using the Maximum Likelihood method and the Tamura–Nei model (MEGA) version 6.0 software to evaluate the evolutionary relationships. The obtained *COI* gene sequences from the identified ticks were deposited in GenBank, and the confirmed *H. anatolicum* sequences were assigned accession numbers PQ613621—PQ613626.

### 2.5. Blood Samples for Blood Parasite Examination

Blood samples were collected from the tail vein of the examined cows into tubes containing ethylenediaminetetraacetic acid (EDTA) and subsequently transported to the laboratory. Thin blood smears were prepared from each sample, fixed with methanol, and stained with Giemsa stain following the protocol described by Feldman et al. (2000) [28]. The stained slides were examined under an oil immersion lens (×100 magnification), and intraerythrocytic parasites were identified according to the criteria described by Soulsby (1982) [29].

### 2.6. Milk Yield Evaluation

Milk samples were collected to assess both the quantity and quality of milk from tick-infested and tick-free (control) cows over a two-month period. Samples were taken twice daily (at 04:00 AM and 04:00 PM) and stored in sterile, leak-proof vials. All sample collections were performed by the same staff member to minimize inter-observer variation. For each cow, daily milk production (liters per cow per day) was recorded for 8 consecutive weeks (tick-infested vs. tick-free controls; morning vs. afternoon). The raw, per-cow, per-milking data for week 1to week 8 are provided in Supplementary Table S4. For all weeks, we then computed weekly group means for each group and milking time; this averaging naturally reduces apparent variability within groups [30]. As expected, aggregation at the group–week level reduces the apparent within-group variability. Control cows, all of the same breed (Jersey) and maintained under standardized feeding, housing, and milking conditions, exhibited stable physiological status throughout the study. In contrast, the tick-infested group displayed greater variability, reflecting physiological stress and individual differences in response to infestation.

To evaluate the relationship between tick infestation and milk yield, a linear regression model was applied. The model used week number (1–8) as the independent variable and mean milk yield (L) as the dependent variable. Separate regression models were fitted for morning and afternoon milk yields in tick-infested cows. The regression equation was expressed as follows:

Y = β0 + β1X + ϵ

where Y represents milk yield (L), X represents the week number, β0 is the intercept, β1 is the slope (change in milk yield per week), and ϵ is the error term.

#### Chemical and Mineral Analysis of Cow’s Milk

Milk composition was determined using samples collected from all cows during the morning milking session over eight weeks. Upon collection, samples were placed into sterile, labeled tubes, stored at 4 °C, and transported to the analytical laboratory within four hours to preserve their physicochemical properties [31].

To assess milk composition, a method was used to measure mineral concentrations, particularly calcium and potassium, using quantitative spectroscopic techniques [30]. Protein and fat contents were determined using a volumetric chemistry analyser, according to standard protocols Supplementary Table S5 [32,33].

SCC was determined using the Direct Microscopic Count (DMC) method. Milk smears were stained with methylene blue, and ten microscopic fields per sample were examined under oil immersion [34]. SCC was calculated using the following formula:

$$\mathrm{SCC}\;(\mathrm{cells}/mL)=\frac{(\mathrm{average}\;\mathrm{number}\;\mathrm{of}\;\mathrm{cells}/\mathrm{field})\times \;\mathrm{microscope}\;\mathrm{factor}\;}{\;\mathrm{Milk}\;\mathrm{volume}\;\mathrm{per}\;\mathrm{smear}\;(mL)}.$$

The reading method for samples was double-blind for both samples (duplicate analysis).

### 2.7. Blood Sampling and Biochemical Measurement

Jugular blood samples were aseptically collected from each cow using 10 mL sterile test tubes and immediately divided into two equal parts. One 5 mL portion was placed in a tube without EDTA for hematological analysis, while the other 5 mL was placed in a tube containing EDTA as an anticoagulant for serum analysis under sterile and controlled conditions. All procedures for blood collection and handling were performed in accordance with established veterinary hematology standards [35].

#### 2.7.1. Serum Blood Biochemistry Analysis

Serum samples were allowed to clot at room temperature for 30–45 min and then centrifuged at 3000 rpm for 10 min. The separated serum was carefully transferred into sterile Eppendorf tubes and stored at −20 °C until further analysis.

Serum biochemical parameters were determined using automated analyzers following the manufacturers’ protocols. The following parameters were measured: liver enzymes (AST and GGT). Renal function markers: creatinine and urea, and electrolytes (calcium (Ca) and potassium (K).

AST, GGT, urea, and creatinine levels were measured using an automated chemistry analyzer (Mindray BS-240) [36]. Serum calcium was also quantified using the BS-200 Chemistry Analyzer (Mindray Medical International Ltd., Shenzhen, China) [37,38], while potassium concentrations were measured using the Ion-Selective Electrode (ISE) method on the Blood Analyzer L platform [39].

All reagents used were of analytical reagent grade, and internal quality control procedures were implemented for each batch to ensure the accuracy, precision, and reproducibility of the results. Serum biochemistry data are provided in Supplementary Table S6. 

#### 2.7.2. Hematological Analysis

Blood samples for hematological examination were collected into tubes containing ethylenediaminetetraacetic acid (EDTA) to prevent coagulation. After gentle inversion to ensure homogeneity, the samples were analyzed immediately to avoid cellular degradation, following the procedure described by [35]. All hematological parameters were measured using the Mindray BC-2800 Vet, an automated veterinary hematology analyzer [40]. The parameters assessed included white blood cell (WBC) count, red blood cell (RBC) count, hemoglobin concentration (HGB), and hematocrit (HCT). All hematological analyses were performed within two hours of sample collection to prevent artifacts and ensure data accuracy. Hematology data are provided in Supplementary Table S7.

### 2.8. Statistical Analysis

Data were analyzed using IBM SPSS Statistics (Version 20.0). The mean, standard deviation (SD), and standard error percentage (SE%) were calculated for all measured variables. The Shapiro–Wilk test was used to assess the normality of the data (*n* = 15 per group); all datasets met the assumption of normal distribution (*p* > 0.05), allowing the use of parametric tests.

Differences between tick-infested and non-infested (healthy) cows in milk yield (morning and evening), blood biochemical parameters (AST, GGT, urea, and creatinine), hematological indices (WBC, RBC, HGB, and HCT), and SCC were evaluated using independent sample *t*-tests. The homogeneity of variances between groups for the same parameters, indicative of biological response consistency, was assessed using F-tests.

A week-by-week comparison of milk yield was performed using two-sample *t*-tests for each week (1–8), and F-tests were conducted to determine differences in variance across weeks within groups. To evaluate changes in milk production over time within each group, one-way ANOVA was applied, followed by Tukey’s HSD post-hoc test to identify significant differences between weeks.

Linear regression analysis was conducted separately for tick-infested and healthy cows to model trends in milk yield over time. Regression coefficients (β), coefficients of determination (R^2^), and associated *p*-values were reported.

All statistical tests were two-tailed, and *p*-values < 0.05 were considered statistically significant. The specific statistical tests applied are indicated in the respective figure legends.

## 3. Results

### 3.1. Morphological Identification of Ticks

A total of 250 adult ticks were collected from cows in Group I (tick-infested group) over the eight-week study period for species-level identification. No ticks were detected or removed from the control group (Group II) during daily inspections. The specimens were curated and examined under a dissecting stereomicroscope and morphologically identified using standard taxonomic keys.

All examined specimens exhibited external morphological features characteristic of the genus *Hyalomma* and were identified as *H. anatolicum*. Species identification was based on a combination of diagnostic traits, including the genital aperture, mouthparts, scutal ornamentation, and configuration of the leg coxae, consistent with published descriptions.

**Figure 1:**
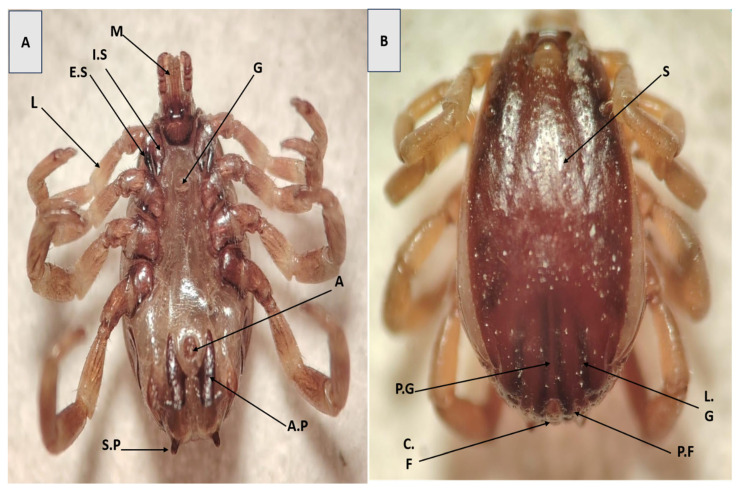
(**A**) Ventral and (**B**) dorsal surfaces of *H. anatolicum* (Male). (M) Mouth part; (L) Leg; (E.S) External spur; (I.S) Internal spur; (G) Genital aperture; (A) Anus; (A.P) Adanal platesas; (S.P) Subanal platesas; (S) Scutum; (P.G) Posteromedium groove; (L.G) Lateral grooves; (C.F) Central festoon; (P.F) Paracentral festoons.

In male specimens, key diagnostic features included a well-defined hexagonal basis capituli, cervical field depression, short lateral grooves, and two posterior ridges on the scutum. Identification was further supported by the alignment of the subanal plates with the adanal plates along their long axis (Figure 1B). The ventral aspect of coxa I displayed prominent internal and external spurs, whereas coxae II and III exhibited only external spurs, and coxa IV lacked clear internal spurs. The mouthparts were robust and anteriorly directed, with a hexagonal basis capituli visible dorsally—another distinguishing feature of *H. anatolicum* (Figure 1A).

**Figure 2:**
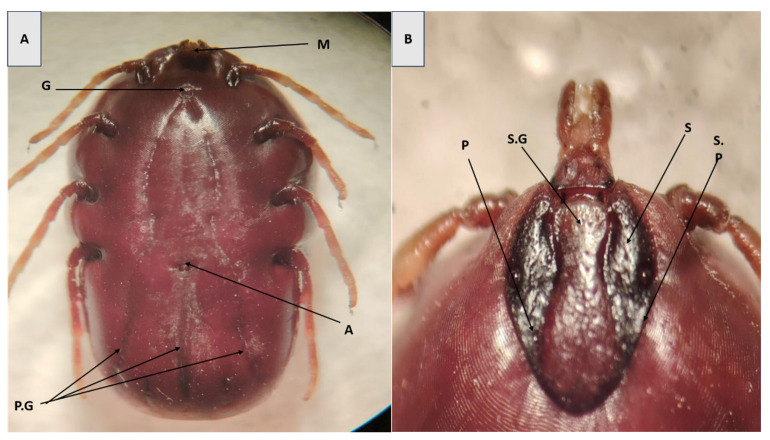
(**A**) Ventral and (**B**) dorsal surfaces of *H. anatolicum* (Female). (M) Mouth part; (G) Genital aperture; (A) Anus; (P.G) Posteromedium groove; (S.G) Scapular grooves; (S) Scutum; (P) Punctation; (S.P) Scutum posterior.

In female specimens, diagnostic traits included a U-shaped genital aperture, steep scutal grooves, pale leg banding, small porose areas, and a hexagonal basis capituli (Figure 2A). The dorsal shield exhibited a complete set of festoons—small, rectangular grooves along the posterior margin of the body—which is a notable characteristic of the genus and aids in species-level identification (Figure 2B). 

### 3.2. Molecular Characterization of Tick Infestations

**Figure 3:**
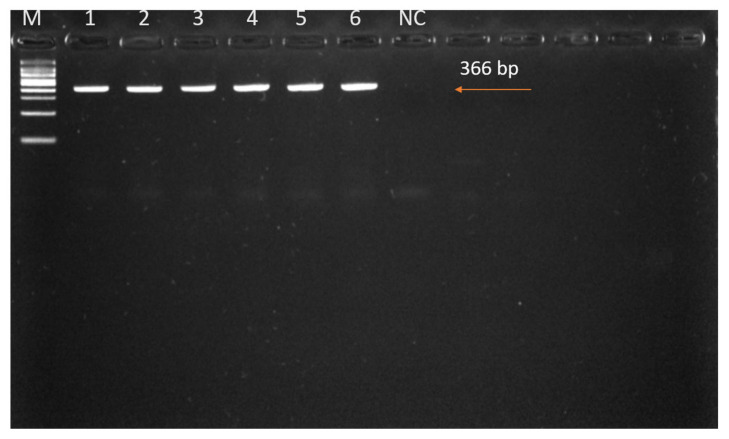
PCR amplification of the mitochondrial *COI* gene from DNA extracted from *Hyalomma* spp. Lane M: 100 bp DNA ladder; Lanes 1–6: amplified tick DNA samples; Lane NC: no-template negative control. The expected amplicon size of ~366 bp is indicated. No bands were observed in the NC lane, confirming the absence of contamination.

We successfully amplified the *COI* gene sequences from six adult tick samples using PCR. One adult tick was selected from each of six different cows (out of the fifteen tick-infested individuals) for molecular identification. These ticks had been morphologically identified as *H. anatolicum* based on consistent diagnostic features observed across the population. DNA extraction and PCR amplification targeting the mitochondrial *COI* gene were conducted to confirm species identity. The PCR amplicon was approximately 366 bp in length (Figure 3), and subsequent sequence analysis validated the morphological identification. The resulting sequences exhibited high similarity to previously reported *H. anatolicum* sequences from the region, as documented in GenBank (accession numbers PQ613621–PQ613626).

### 3.3. Phylogenetic Analysis

**Figure 4:**
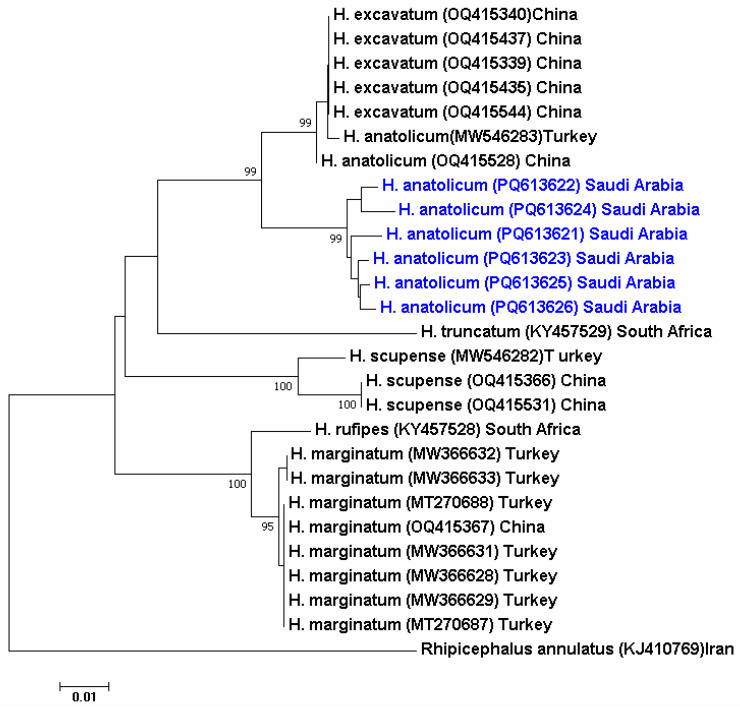
The phylogenetic tree using the NJ method in MEGA version 6.0 based on the mitochondrial *COI* gene sequences of *Hyalomma* spp. Sequences generated in this study (PQ613621–PQ613626) are highlighted in blue and represent *H. anatolicum* specimens collected in Saudi Arabia. These sequences clustered closely with reference *H. anatolicum* isolates from Turkey and China, supporting species-level identification. *Rhipicephalus annulatus* (KJ410769) was used as the outgroup. Bootstrap values > 70% are shown at the branch nodes.

A Neighbor-Joining (NJ) tree was initially constructed based on the mitochondrial *COI* gene sequences obtained from the six tick samples analyzed in this study. The resulting sequences showed a high degree of similarity (>99%) with *H. anatolicum* reference sequences from Turkey (GenBank accession no. MW546283), China (OQ415528), and also *H. excavatum* (Figure 4). 

### 3.4. Hemoparasite Detection

**Figure 5:**
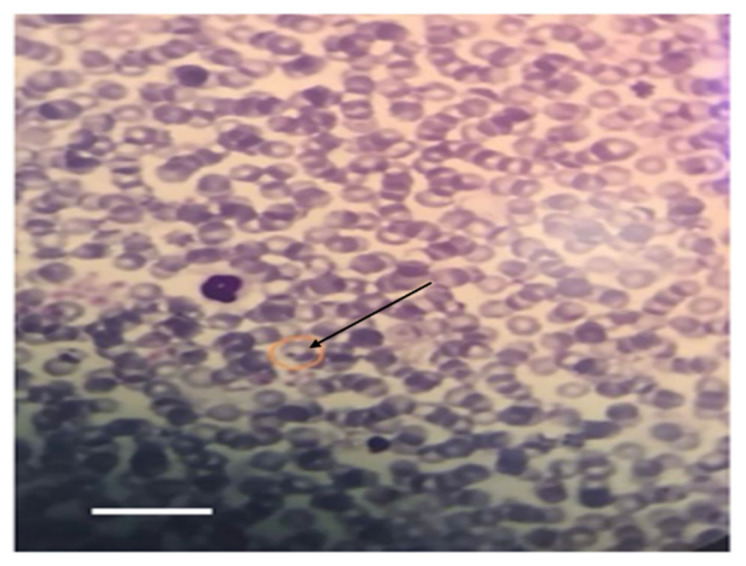
Giemsa-stained blood smear from a tick-infested cow showing an intraerythrocytic piroplasm (black arrow) consistent with *Theileria* spp. infection. Parasites were detected in 2/15 (13.3%) infested cows; none were observed in smears from healthy controls. Scale bar = 10 μm.

Microscopically, intraerythrocytic piroplasms morphologically consistent with *Theileria* spp. were detected in Giemsa-stained blood smears from 2 out of the 15 tick-infested cows. In contrast, blood smears from the healthy control group showed no detectable hemoparasites (Figure 5).

### 3.5. The Average Variation in the Production of Milk

#### 3.5.1. Weekly Trends in Milk Yield

**Figure 6:**
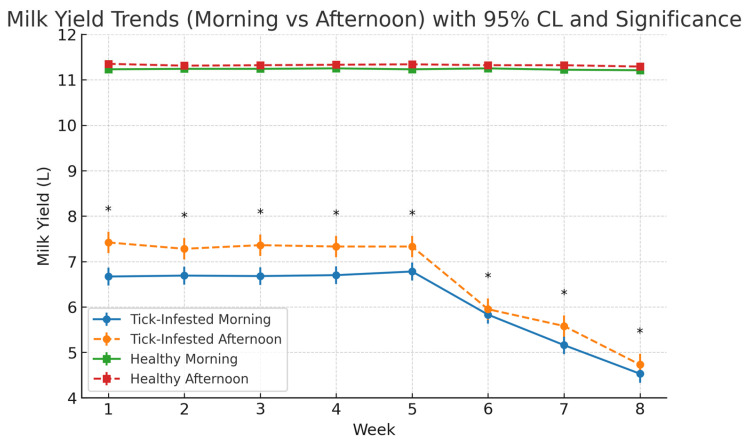
Weekly milk yield (L) in tick-infested and healthy cows during morning and afternoon milking sessions over an 8-week period. Each point represents the mean milk yield per session per week (n = 15 cows per group). Error bars indicate 95% confidence intervals. Asterisks denote statistically significant differences between morning and afternoon yields within the tick-infested group for each week (*p* < 0.0001, paired *t*-test).

Figure 6 presents the weekly monitoring of milk yield over an eight-week period in tick-infested and healthy (control) cows, with measurements taken independently during morning and afternoon milking sessions. In healthy cows, milk production remained stable throughout the study. Morning yields ranged from 11.21 L to 11.25 L, with an average of 11.23 ± 95% CL, while afternoon yields ranged from 11.29 L to 11.35 L, averaging 11.32 ± 95% CL. No significant weekly variation was observed. The relatively narrow range of yields in the control group (11.0–11.3 L) reflects standardized breed, feeding, and housing conditions, as well as the use of weekly group means, which reduces apparent variability compared to the tick-infested group.

In contrast, tick-infested cows exhibited a progressive decline in milk yield beginning after week 5. Morning milk yield declined from 7.42 L in week 1 to 4.73 L by week 8 (overall mean: 6.62 ± 95% CL), while afternoon milk yield decreased from 6.67 L to 4.53 L over the same period (overall mean: 6.13 ± 95% CL) (Table 1). Weekly averages revealed a consistent diurnal pattern, with afternoon yields exceeding morning yields in both groups across all weeks. Paired *t*-tests confirmed these differences were statistically significant (*p* < 0.0001) in both the tick-infested group (*t* = 29.80–43.26) and the control group (*t* = 17.01–23.68). See Supplementary Table S8 for weekly group means of milk yield.”

Linear regression models for the tick-infested group indicated strong linear declines in both morning (*R*^2^ = 0.71) and afternoon (*R*^2^ = 0.77) yields across the study period, showing that week of lactation explained a substantial proportion of the variance in milk yield. These findings suggest that progressive tick burden exerted a sustained and measurable negative influence on milk productivity in both milking sessions.

#### 3.5.2. Effect of Tick Infection on Milk Components

Tick infestation had a significant impact on milk composition and SCC, as detailed in Table 1. Marked differences were observed between tick-infested and healthy cows across all measured parameters.

Milk calcium levels were significantly lower in infested cows (820.85 ± 2.25 mg/L) compared to healthy cows (1178.45 ± 2.66 mg/L), with the difference being highly significant (*p* < 0.0001). Similarly, potassium concentrations were substantially reduced in tick-infested cows (939.25 ± 6.68 mg/L) versus healthy controls (1587.36 ± 0.87 mg/L; *p* < 0.0001).

Protein content was significantly lower in the milk of tick-infested cows (1.34 ± 0.02%) than in healthy cows (3.81 ± 0.06%; *p* < 0.0001). A comparable pattern was observed for fat content, with infested cows yielding 1.15 ± 0.04% compared to 3.48 ± 0.09% in healthy animals (*p* < 0.0001).

In addition to these compositional changes, SCC was significantly elevated in tick-infested cows (580,000 ± 120,000 cells/mL) compared to healthy cows (310,000 ± 95,000 cells/mL; *p* < 0.01). The elevated SCC suggests ongoing inflammatory processes within the mammary gland, potentially indicating subclinical mastitis or a systemic immune response triggered by tick infestation.

Overall, these findings demonstrate that tick infestation negatively affects both the nutritional quality and hygienic status of milk. This is reflected not only in the reduced concentrations of essential minerals and macronutrients but also in elevated indicators of mammary inflammation (Table 1).

### 3.6. Serum Biochemical

The serum biochemical profile of tick-infested cows showed significant alterations compared to healthy controls, as presented in Table 2. Liver enzyme levels were markedly elevated in the infested group, with AST and gamma-glutamyl transferase (GGT) recorded at 140.5 ± 1.2 IU/L and 33.5 ± 1.1 IU/L, respectively, compared to 41.5 ± 1.3 IU/L and 13.1 ± 0.8 IU/L in the control group (*p* < 0.0001).

Similarly, renal function markers showed significant increases in tick-infested cows. Serum creatinine levels reached 8.0 ± 0.2 mg/dL and urea 32.2 ± 1.0 mg/dL, while in healthy controls these values were 0.9 ± 0.05 mg/dL and 14.0 ± 0.9 mg/dL, respectively (*p* < 0.0001).

Electrolyte balance was also affected. Mean serum calcium levels were significantly reduced in tick-infested cows (7.50 ± 0.9 mg/dL) compared to healthy animals (12.40 ± 0.6 mg/dL; *p* < 0.0001). In contrast, serum potassium levels were significantly lower in infested cows (2.44 ± 0.17 mmol/L) compared to the control group (4.45 ± 0.63 mmol/L; *p* < 0.0001).

These findings indicate that tick infestation induces marked metabolic disruptions, reflected by liver dysfunction, renal stress, and electrolyte imbalances (Table 2).

### 3.7. Hematological Results

The hematological profile further indicated systemic effects associated with tick infestation. WBC count was significantly elevated in tick-infested cows (25.5 ± 0.9 × 10^3^/µL) compared to non-infested controls (17.6 ± 0.7 × 10^3^/µL; *p* < 0.0001). A pronounced increase in red blood cell (RBC) count was observed in the infested group (19.1 ± 0.6 × 10^6^/µL) versus controls (8.5 ± 0.5 × 10^6^/µL; *p* < 0.0001).

Despite the elevated RBC count, severe anemia was evident in the infected cows, as reflected by a significantly reduced HGB concentration (3.0 ± 0.2 g/dL) compared to the control group (6.4 ± 0.3 g/dL; *p* < 0.0001). HCT was also slightly but significantly lower in the tick-infested cows (45.7 ± 0.5%) than in healthy controls (47.2 ± 0.4%; *p* < 0.0001) (Table 3).

## 4. Discussion

Accurate morphological identification of *Hyalomma* species remains crucial for reliable species assignment in field-based tick surveillance. In this study, all adult ticks collected from dairy cattle in Hafar Al-Batin, Eastern Saudi Arabia, were morphologically identified as *H. anatolicum* based on standard taxonomic keys. Diagnostic features included a U-shaped genital aperture, prominent scapular grooves, pale annulated legs, well-defined coxal spurs, and festoons in female ticks, while male ticks exhibited cervical depressions, an ornamented scutum, and parallel adanal and subanal plates. These characteristics are consistent with classical morphological descriptions of *H. anatolicum* reported across the Middle East and North Africa [41,42,43].

Similar morphological profiles have been documented in regional studies: in the United Arab Emirates, *H. anatolicum* and *H. dromedarii* were distinguished by features such as genital aperture shape and coxal spur structure [43]; in Iraq, Al-Hamadan et al. [44] observed nearly identical morphological features in adult ticks from small ruminants; and in Iran, In Iran, Hosseini-Chegeni et al. [45] confirmed the diagnostic utility of ventral plate alignment and the prominence of the cervical (scapular) grooves for separating *Hyalomma* species. In Turkey, Aktaş et al. [46] (eastern Turkey) and Iça et al. [47] (central Anatolia) identified *Hyalomma anatolicum* from cattle using standard morphological keys coxae I, IV spur development, cervical (scapular) grooves, and the shape, alignment of the male adanal subanal plates (genital plate region).

Over 15 ixodid tick species have been recorded in Saudi Arabia, including *H. arabicum*, *H. turanicum*, and *H. anatolicum* [9,23,48]. In the present study, *H. anatolicum* was again identified as the dominant species, consistent with earlier reports highlighting its ecological adaptation to arid environments [49,50,51,52].

Geographic assignment to *Hyalomma anatolicum* is well supported for the Hafar Al-Batin sampling site. Although *H. excavatum* has not been specifically reported from Hafar Al-Batin, evidence from adjacent, ecologically comparable areas of Eastern Saudi Arabia indicates that it is rare or absent. In Al-Ahsa (Eastern Region), a large livestock survey (n = 5320 ticks) recorded *H. anatolicum*—including specimens historically designated as *H. anatolicum excavatum*—as >32% of collections and did not treat *H. excavatum* as a separate species [53]. A multi-province cattle study likewise found frequent *H. anatolicum* and *H. impeltatum* but only a single *H. excavatum* specimen [54], and camel collections in Riyadh Province showed *H. excavatum* constituted just 0.3% (1/296) compared with dominant *H. anatolicum* and *H. dromedarii* [5]. Region-wide species-distribution modelling also identifies *H. dromedarii* and *H. anatolicum* as the principal camel-associated *Hyalomma* in arid central/eastern Saudi Arabia [55]. Taken together, the established presence of *H. anatolicum* at Hafar Al-Batin and the consistent rarity of *H. excavatum* in comparable settings provide strong geographic support for assigning our material to *H. anatolicum*.

The use of PCR and sequencing to confirm morphological identification is a reliable method, as morphological features alone can often result in misidentification due to intraspecific variation. This is crucial because molecular confirmation improves morphological identification, which can be ambiguous due to intraspecific differences. A phylogenetic tree was constructed based on NJ analysis of the *COI* DNA sequence for the six study samples. Based on the generated phylogenetic tree, which revealed >99% sequence similarity (GenBank accessions: PQ613621–PQ613626) with *H. anatolicum* isolates from Turkey and China [56,57], and also *H. excavatum*.

Although phylogenetic analysis revealed close similarities to both *H. anatolicum* and *H. excavatum*, morphological examination confirmed the specimens’ identity as *H. anatolicum*. Diagnostic features included big size, elongated body, long palps, brown scutum, eyes, bifid spurs on coxae I, and prominent leg annulations. These characteristics are compatible with established taxonomic keys. In the studies conducted by Abbasi et al. [58] (2017) and Ali et al. (2024) [59], they showed that males of *H. anatolicum* have large, equal coxa spurs, but *H. excavatum* has a distinct arch and raised ridges on the scutal caudal area. Furthermore, the collection sites are in the known endemic range of *H. anatolicum* in hot and dry climates of the Mediterranean, Middle East, Central Asia, and parts of Africa, with few records of *H. excavatum* in this area [60,61]. Key separating characters for *H. anatolicum* versus *H. excavatum* are summarized in Supplementary Table S9.

In the United Arab Emirates, both *H. anatolicum* and *H. dromedarii* have been identified using molecular markers such as *COI* and *16S rRNA* genes, reinforcing the regional applicability of these loci for tick species confirmation [62]. Phylogenetic analysis in our study clearly clustered *H. anatolicum* distinctly from other *Hyalomma* species and from *Rhipicephalus annulatus*, demonstrating the specificity and robustness of the *COI* gene as a molecular marker for tick species discrimination. This finding aligns with previous studies confirming the efficacy of the *COI* gene in resolving phylogenetic relationships among ixodid ticks, including those of the *Hyalomma* and *Rhipicephalus* genera [63,64]. Similar integrative approaches have been successfully implemented in regional tick studies in Egypt, Lebanon, and Iraq, enhancing the reliability of species-level identification and supporting targeted vector control programs [65,66]. While such dual-method frameworks are well established in some MENA countries, national-scale surveillance systems remain under development in others, including Iraq, where molecular-based identification has been recently initiated using mitochondrial markers [67].

*H. anatolicum* is a recognized vector of *Theileria annulata*, the causative agent of tropical theileriosis—a disease of major economic importance in Saudi Arabia and other Arab countries [44,68]. Most prior molecular and parasitological surveys on *Theileria* infections have focused on the central and western regions of the country. For instance, a molecular study sampling cattle from Riyadh, Al-Kharj, Al-Hasa, and Al-Qassim reported 1.9% prevalence of *T. annulata* and 0.6% for *T. ovis* in 362 animals, with cattle in Riyadh showing the highest infection rates [54]. Additionally, older microscopy-based work documented *Theileria* spp. as one of the most common hemoparasites affecting cattle within central regions such as Qassim [69]. These findings collectively highlight both the fragmented nature of previous studies and the need for molecular surveys in eastern Saudi Arabia.

To our knowledge, this is the first report from Eastern Saudi Arabia linking tick infestation to both decreased milk yield and altered milk composition in dairy cows. Our data show a clear decline in production among tick-infested cows—from 7.42 L in week 1 to 4.73 L by week 8—while non-infested animals maintained a stable output. These findings align with previous studies reporting milk yield reductions of 25–40% in tick-infested cattle. For example, de Castro (1997) reported that heavy tick burdens in dairy cattle can substantially depress milk yield and overall productivity in developing-country systems [70], while Bondan. (2018) documented significant yield loss and udder damage associated with tick bites in Brazil [71]. Similarly, Norval et al. (1997) longitudinal studies from Zimbabwe reported consistent declines in milk yield under chronic tick infestation, with significant losses across lactation [72]. Although such quantitative studies are lacking in Saudi Arabia, our results provide new regional evidence of the economic impact of tick infestation in arid dairy production systems. Hurtado and Giraldo-Ríos (2018) [12] also emphasized the broader economic and health impact of ticks on ruminant productivity. Supporting this trend, Satti et al. (2021) [73] reported a significant burden of tick-borne diseases—such as anaplasmosis and babesiosis—on cattle health and productivity in Khartoum State, Sudan, underscoring regional similarities in the physiological and economic consequences of infestation.

The observed decline in milk production in tick-infested cows is likely multifactorial. These cows had visible tick infestations at the start of the experiment, as confirmed by veterinary examination, while control cows—though from the same free-grazing herd—were free of ticks. The initially lower milk yield in the infested group reflects early physiological impacts of tick burden. The sharper decline observed between weeks 5 and 8 is likely due to cumulative effects, including persistent blood loss, localized inflammation and skin lesions, chronic systemic inflammation, and stress-induced metabolic disruption. Although adult ticks were removed daily for monitoring, no acaricidal treatment was applied, and immature tick stages likely reattached, compounding the parasitic load in later weeks. Reduced feed intake, physiological stress, and metabolic imbalance, as described by Azambuja et al. (2020) [74], likely contributed to the observed yield loss. Notably, afternoon milk yields were consistently higher than morning values across all weeks, potentially reflecting diurnal variation in feed intake and energy metabolism [75,76]. These findings are consistent with studies that emphasize the detrimental impact of parasitic pressure on both milk yield and composition [77,78].

This study also provides the first documentation in Eastern Saudi Arabia of tick infestation impacting milk mineral composition. We observed substantial reductions in calcium (30.3%) and potassium (40.8%) levels in milk from infested cows. These results echo findings from Egypt, where Noha et al. (2024) reported significantly decreased calcium and zinc concentrations in milk from cattle infested with ticks and infected with blood parasites, compared to non-infested controls [79]. Calcium is essential for mammary gland function, and its depletion may reflect systemic metabolic stress or hormonal disruption [80]. Similar reductions in milk calcium have been reported in Pakistan and attributed to endocrine imbalance and udder dysfunction [81]. Potassium, a key regulator of osmotic balance and enzyme function, was also markedly reduced, likely due to inflammation-related electrolyte shifts [82]. These changes may impair milk quality and pose potential risks to consumer safety.

Milk protein and fat contents were reduced by 64.8% and 66.9%, respectively, in tick-infested cows. These reductions support previous findings that parasitic stress—particularly due to ectoparasites like ticks can impair mammary gland metabolism and inhibit macronutrient synthesis in lactating animals. Such alterations in milk composition have critical implications for nutritional quality, consumer safety, and dairy product yield, especially in rural regions with limited veterinary infrastructure or quality control measures [83,84].

SCC was significantly elevated (mean = 580 × 10^3^/mL) in infested cows compared to healthy controls (mean = 310 × 10^3^/mL), representing an 87.1% increase. Elevated SCC is a well-recognized marker of subclinical mastitis and inflammation [17,18], and similar trends have been reported in cows exposed to ectoparasites [85]. The increase in SCC may result from immunosuppressive effects of tick-borne pathogens (*Babesia*, *Anaplasma*, *Theileria*) and associated secondary infections [86,87].

The marked alterations in serum biochemistry observed in tick-infested cows highlight the systemic physiological burden imposed by chronic parasitism. In our study, AST and GGT levels increased by 238% and 156%, respectively, relative to healthy controls. These enzymes are classical indicators of hepatocellular damage and biliary stress, suggesting significant liver involvement in response to the prolonged inflammatory insult induced by tick feeding. This aligns with previous reports by El-Tarabany et al. (2018), who observed elevated hepatic enzymes in Holstein cows infected with blood parasites, attributing these elevations to hepatic congestion and oxidative stress [84].

Moreover, serum urea and creatinine concentrations were significantly elevated (130% and 789%, respectively), strongly indicating compromised renal clearance or intensified protein catabolism. These findings are consistent with those of Saleem et al. (2020) in Pakistan, who reported increased urea and creatinine levels in *Babesia*-infected cattle, suggesting nephrotoxic effects due to systemic inflammation and hemolysis [88].

Importantly, we also observed significant electrolyte imbalances in tick-infested cows for which serum calcium and potassium were lower than in healthy controls (calcium: 7.50 ± 0.90 vs. 12.40 ± 0.60 mg/dL; potassium: 2.44 ± 0.17 vs. 4.45 ± 0.63 mmol/L). Hypocalcaemia and hypokalaemia in this context likely reflect mineral sequestration due to tissue injury, inflammation-induced loss, or impaired hormonal regulation (e.g., parathyroid hormone). A similar biochemical profile was reported by Goswami et al. (2024) in naturally tick-infested cows in northern India, where significant reductions in calcium and phosphorus levels were associated with parasitic burden [89].

Collectively, these biochemical changes provide valuable diagnostic insight into the multi-organ stress response elicited by ticks. They suggest that hepatic, renal, and metabolic pathways are profoundly impacted during chronic ectoparasitic infestations. Such findings underscore the importance of incorporating routine serum biochemistry into herd health surveillance, especially in regions with endemic tick exposure.

The haematological profile of tick-infested cows further underscores the systemic stress associated with prolonged ecto-parasitism. Notably, WBC counts were markedly elevated (25.50 ± 0.90 × 10^3^/µL) compared to healthy controls, indicating a robust immune response to continuous tick exposure, blood loss, and potential pathogen transmission, such as *Theileria* or *Babesia* spp. This leukocytosis is consistent with the inflammatory state induced by parasitic infestation and is a common finding in tick-borne infections [89].

Interestingly, we also observed increased RBC counts (19.10 ± 0.60 × 10^6^/µL), a less commonly reported feature. This may reflect compensatory erythrocytosis triggered by hypoxic stress, dehydration, or adrenergic stimulation due to inflammation. Similar trends were documented by Kim et al. (2024), who noted elevated RBC values in tick-infested cattle possibly due to splenic contraction and stress-induced haemoconcentration [90].

However, despite the elevated RBC count, HGB and HCT levels were significantly reduced (3.00 ± 0.20 g/dL and 45.70 ± 0.50%, respectively), indicating either non-functional erythrocytes, hemolysis, or anemia of chronic disease (ACD)—a condition commonly associated with parasitic infections, where inflammation suppresses erythropoiesis and iron utilization. These findings are consistent to report from Nazifi et al. (2012), these findings are consistent with reports of hemolytic anemia in bovine anaplasmosis and babesiosis conditions in which hemoglobin and hematocrit fall markedly, reflecting hemolysis and impaired erythropoiesis. [91].

Altogether, these haematological alterations reflect a complex physiological adaptation to parasitic pressure, characterized by immune activation, erythroid stress, and metabolic imbalance, reinforcing the need for early tick control to mitigate systemic pathology.

By integrating milk production parameters with hematological and biochemical indicators, this study offers a holistic assessment of the physiological burden imposed by tick infestation in dairy cattle. These results highlight the pressing need for holistic tick control strategies that encompass prudent use of acaricides, surveillance by veterinarians, specific mineral supplementation, and nutritional support. Implementing such measures is essential to reduce economic losses, safeguard animal welfare, and improve milk quality and safety in arid and semi-arid dairy systems.

## 5. Conclusions

This study confirms the presence of *H. anatolicum* in dairy cattle from Hafar Al-Batin, Eastern Saudi Arabia, through both morphological and molecular identification. Tick infestation significantly reduced milk yield and altered milk composition, highlighting its substantial impact on dairy productivity and cow health.

Although limited by sample size and study duration, the findings provide important baseline data and emphasize the need for integrated tick management strategies. Future research should involve larger cohorts, molecular screening for co-infections, and analysis of tick genetic variation to better understand long-term effects.

Regional efforts should prioritize geospatial risk mapping and shared tick-pathogen databases to strengthen surveillance and control.

## Figures and Tables

**Table 1:** Effect of tick infestation on milk yield and chemical composition in dairy cows over the eight-week study period. Values are presented as mean ± standard error (SE). Percentage differences are calculated relative to the healthy (control) group.

Parameter	Tick-Infested Cows (Mean ± SE)	Healthy Cows (Mean ± SE)	% Difference	*p*-Value
Daily Milk Yield—Morning (L)	6.62 ± 0.37	11.23 ± 0.005	↓ 41.0%	<0.0001
Daily Milk Yield—Afternoon (L)	6.13 ± 0.31	11.32 ± 0.006	↓ 45.9%	<0.0001
Calcium (mg/L)	820.85 ± 8.75	1178.45 ± 10.30	↓ 69.7%	<0.0001
Potassium (mg/L)	939.25 ± 5.90	1587.36 ± 3.35	↓59.2%	<0.0001
Protein (%)	1.34 ± 0.02	3.81 ± 0.06	↓ 64.8%	<0.0001
Fat (%)	1.15 ± 0.04	3.48 ± 0.09	↓ 66.9%	<0.0001
SCC (×10^3^/mL)	580 ± 120	310 ± 95	↑ 87.1%	<0.01

Values are mean ± SE. % difference = 100 × [(tick-infested − healthy) ÷ healthy]. Arrows indicate the direction for the tick-infested group relative to controls: ↓ lower mean parameter value; ↑ higher mean parameter value. *p*-values are two-tailed.

**Table 2:** Serum biochemical parameters in tick-infested and healthy dairy cows (n = 15 per group). Values are presented as mean ± standard error (SE). Percentage differences are calculated relative to the healthy (control) group.

Parameter	Tick-Infested Cows (Mean ± SE)	Healthy Cows (Mean ± SE)	% Difference	*p*-Value
AST (IU/L)	140.50 ± 1.20	41.50 ± 1.30	↑ 238.6%	<0.0001
Creatinine (mg/dL)	8.00 ± 0.20	0.90 ± 0.05	↑ 788.9%	<0.0001
Urea (mg/dL)	32.20 ± 1.00	14.00 ± 0.90	↑ 130.0%	<0.0001
GGT (IU/L)	33.50 ± 1.10	13.10 ± 0.80	↑ 155.7%	<0.0001
Calcium (mg/dL)	7.50 ± 0.90	12.40 ± 0.60	↓ 39.5%	<0.0001
Potassium (mmol/L)	2.44 ± 0.17	4.45 ± 0.63	↓ 45.2%	<0.0001

Values are mean ± SE. % difference = 100 × [(tick-infested − healthy)/healthy]. Arrows indicate the direction for the tick-infested group relative to healthy controls: ↓ lower mean value; ↑ higher mean value. *p*-values are two-tailed. This table presents the mean ± standard error (SE) of serum biochemical parameters in tick-infested and healthy cows. The percentage difference and *p*-value are shown for each parameter between the two groups. Significant differences (*p* < 0.0001) were observed in all parameters, with tick-infested cows showing significantly higher levels of AST, creatinine, urea, and GGT, and significantly lower levels of calcium and potassium compared to healthy cows.

**Table 3:** Hematological parameters in tick-infested and healthy dairy cows (n = 15 per group). Values are presented as mean ± standard error (SE). Percentage differences are calculated relative to the healthy (control) group.

Parameter	Tick-Infested Cows (Mean ± SE)	Healthy Cows (Mean ± SE)	% Difference	*p*-Value
WBC (×10^3^/µL)	25.50 ± 0.90	17.60 ± 0.70	↑ 44.9%	<0.0001
RBC (×10^6^/µL)	19.10 ± 0.60	8.50 ± 0.50	↑ 124.7%	<0.0001
HGB (g/dL)	3.00 ± 0.20	6.40 ± 0.30	↓ 53.1%	<0.0001
HCT (%)	45.70 ± 0.50	47.20 ± 0.40	↓ 3.2%	<0.0001

Values are mean ± SE. % difference = 100 × [(tick-infested − healthy)/healthy]. Arrows indicate the direction for the tick-infested group relative to healthy controls: ↓ lower mean value; ↑ higher mean value. *p*-values are two-tailed.

## Data Availability

The final assembled sequences were added to the GenBank database with accession numbers [PQ613621-26].

## Supplementary Materials

The following supporting information can be downloaded at: https://www.mdpi.com/article/10.3390/pathogens14090883/s1, Table S1: Regional *Hyalomma* distribution summary (Saudi Arabia & UAE); Table S2. Tick counts per cow, daily by week (Days 1–7 with weekly subtotal). (Tables S2.1week1 to S2.8_Week 8 &Table S2 in text.); Table S3. *Tick* counts—weekly totals per cow and grand total (n = 250 adult ticks); Table S4. Milk yield per cow, per-milking records (04:00 & 16:00) for Weeks 1–8 (tick-infested vs. healthy); Table S5. Chemical composition of milk (mean ± SE); Table S6. Serum biochemical parameters (mean ± SE); Table S7. Hematology parameters by week (mean ± SE); Table S8. Milk yield, weekly group means for Weeks 1–8 (04:00 vs. 16:00; mean ± SE); Table S9. Morphology comparison, *H. anatolicum* vs *H. excavatum* (key separating traits).
